# Investigative study into whether an insect repellent has virucidal activity against SARS-CoV-2

**DOI:** 10.1099/jgv.0.001585

**Published:** 2021-04-23

**Authors:** Sophie J. Smither, Lin S. Eastaugh, James S. Findlay, Thomas R. Laws, Stephen N. Marriott, Stuart Notman, Lyn M. O’Brien, Amanda L. Phelps, Mark Richards, David Ulaeto, Pat Watts, Mark S. Lever, Norman Govan

**Affiliations:** ^1^​ Chemical, Biological and Radiological Division, Defence Science and Technology Laboratory (Dstl), Porton Down, Wiltshire, SP4 0JQ, UK

**Keywords:** anti-viral activity, Citriodiol, insect repellent, SARS-CoV-2, skin

## Abstract

A small-scale study with Mosi-guard Natural spray, an insect repellent containing Citriodiol, was performed to determine if it has virucidal activity against SARS-CoV-2. A liquid test examined the activity of the insect repellent and the individual components for virucidal activity. A surface contact test looked at the activity of the insect repellent when impregnated on a latex surface as a synthetic skin for potential topical prophylactic application. Both Mosi-guard Natural spray and Citriodiol, as well as other components of the repellent, had virucidal activity in the liquid contact test. On a latex surface used to simulate treated skin, the titre of SARS-CoV-2 was less over time on the Mosi-guard Natural-treated surface but virus was still recovered.

Mosi-guard Natural is an insect repellent containing Citriodiol, ethanol and isopropanol. Citriodiol is a commercial distillation of Eucalyptus citriodora oil, whose major component is reported to be p-menthane-3,8-diol (PMD), alongside other constituents [1]. Several patents related to PMD noted that ‘surprisingly’ PMD possesses antiviral properties including activity against the original Severe Acute Respiratory Syndrome Coronavirus (SARS-CoV, Urbani isolate from the 2003 SARS outbreak) [2]. The patents report PMD, tested at a range of concentrations and contact times, resulted in a maximum 2.5 log_10_ reduction in titre of SARS-CoV [2]. We determined if Mosi-guard Natural insect repellent had virucidal activity against SARS-CoV-2, the cause of the COVID-19 pandemic.

SARS-CoV-2 England-2 isolate was provided by Public Health England. Passage 3 material was grown in Vero C1008 cells and enumerated by TCID_50_ assay as previously described [3].

Mosi-guard Natural pump spray and Citriodiol (both Citrefine, Leeds, UK) were used as provided and were tested alongside aqueous solutions of ethanol and isopropanol (Sigma-Aldrich, Gillingham, UK).

To test virucidal activity of Mosi-guard Natural and its main components (representative of the upper range indicated in the spray [4]), 25 µl of the test component (either ethanol, isopropanol, Mosi-guard Natural pump spray or Citriodiol) was added to 25 µl SARS-CoV-2 stock (at 3×10^6^ TCID_50_ ml^−1^) in a polystyrene 24-well culture plate (Corning Costar, Flintshire, UK). For positive controls and to determine the maximum virus recovery, 25 µl tissue culture medium (TCM) was added to 25 µl SARS-CoV-2 stock. As negative controls, 25 µl of the test components was added to 25 µl TCM (no virus). All samples were tested in triplicate. After 1 min, 1 ml=TCM was added, samples were vortexed twice for 10 s and then centrifuged at 14 000 r.p.m. for 10 mins. After centrifugation the liquid was discarded and 1 ml fresh TCM was added to the tube. This was repeated twice. After the final centrifugation, 2 ml TCM was added to the tube and a TCID_50_ assay performed on each sample in duplicate. The centrifugation steps were required to dilute the chemical components that would otherwise cause toxicity in the cell-culture-based enumeration assay, and is similar to methods described previously [5]. Toxicity testing was performed prior to experiments with virus and three washes were shown to be required for Mosi-guard Natural and Citriodiol. To maintain consistency, all samples were subject to the same number of washes.

After the TCID_50_ assay was performed the remainder of each replicate sample was pooled and inoculated onto confluent T12.5 flasks, which were passaged twice as described previously [5]. In a parallel test, 90 µl Mosi-guard Natural spray was added to 10 µl concentrated SARS-CoV-2 (at 1×10^8^ TCID_50_ ml^−1^) for 1 min and processed as above to test Mosi-guard Natural spray at a final concentration of 90 %.

To test the efficacy of Mosi-guard Natural spray on a surface, a latex surface (Hygenic, Akron, OH, USA) of medical grade latex, Hytone HPN-09814 variant, 42’ wide, 0.020’ thick to simulate skin was treated with Mosi-guard Natural using a SprayCraft SP50K air brush and cut into discs, which were placed in a 24-well cell-culture plate. A second 24-well cell-culture plate was air brush-sprayed with Mosi-guard Natural. Untreated discs of latex and untreated 24-well plates were used as surface control samples to determine the natural decay over time of SARS-CoV-2. Approximately 1 h after the Mosi-guard Natural-treatment of surfaces, a single 10 µl droplet of concentrated SARS-CoV-2 was applied to each of the four surfaces with no further spreading. TCM only was also applied as a no virus control. Immediately after virus addition and at 30, 60, 120, 180 and 240 mins after virus addition, triplicate virus samples and one no-virus control were recovered and processed for enumeration and cell-culture passage as described above.

Statistical analysis was performed in Graphpad PRISM v8. For initial tests looking at individual components, statistical significance was determined by one-way ANOVA and Dunnett’s tests or a *T* test with a Welch correction. For the time-course study, linear regression analysis on the logarithm-transformed data was used to model the data. Analysis of covariance was used to compare data-sets. Further details on the methods used can be found online [6].

In the basic liquid test, the mean titre of recovered SARS-CoV-2 and TCM controls was 1.88×10^4^ TCID_50_ ml^−1^ whilst SARS-CoV-2 mixed with other components resulted in reduced titres (Fig. 1a). Mosi-guard Natural and Citriodiol, both tested at 50 % v/v, reduced the titre of virus that could be recovered by 2.4 log_10_ and 3.2 log_10_ respectively, however, viable virus was still recovered. Isopropanol alone (tested at 20 % v/v) had the least activity against the virus with only a tenfold reduction in viable virus recovered. Ethanol, tested at 40 % (v/v) final concentration resulted in no viable virus being recovered by TCID_50_ assay (equivalent to a 4.2 log_10_ reduction in titre) but infection did occur in the second cell passage indicating low levels of virus remained. The combination of ethanol and isopropanol (at final concentrations of 20 and 10 % v/v, respectively) resulted in low levels of virus being recovered and flasks were also positive for infection.

**Fig. 1.**
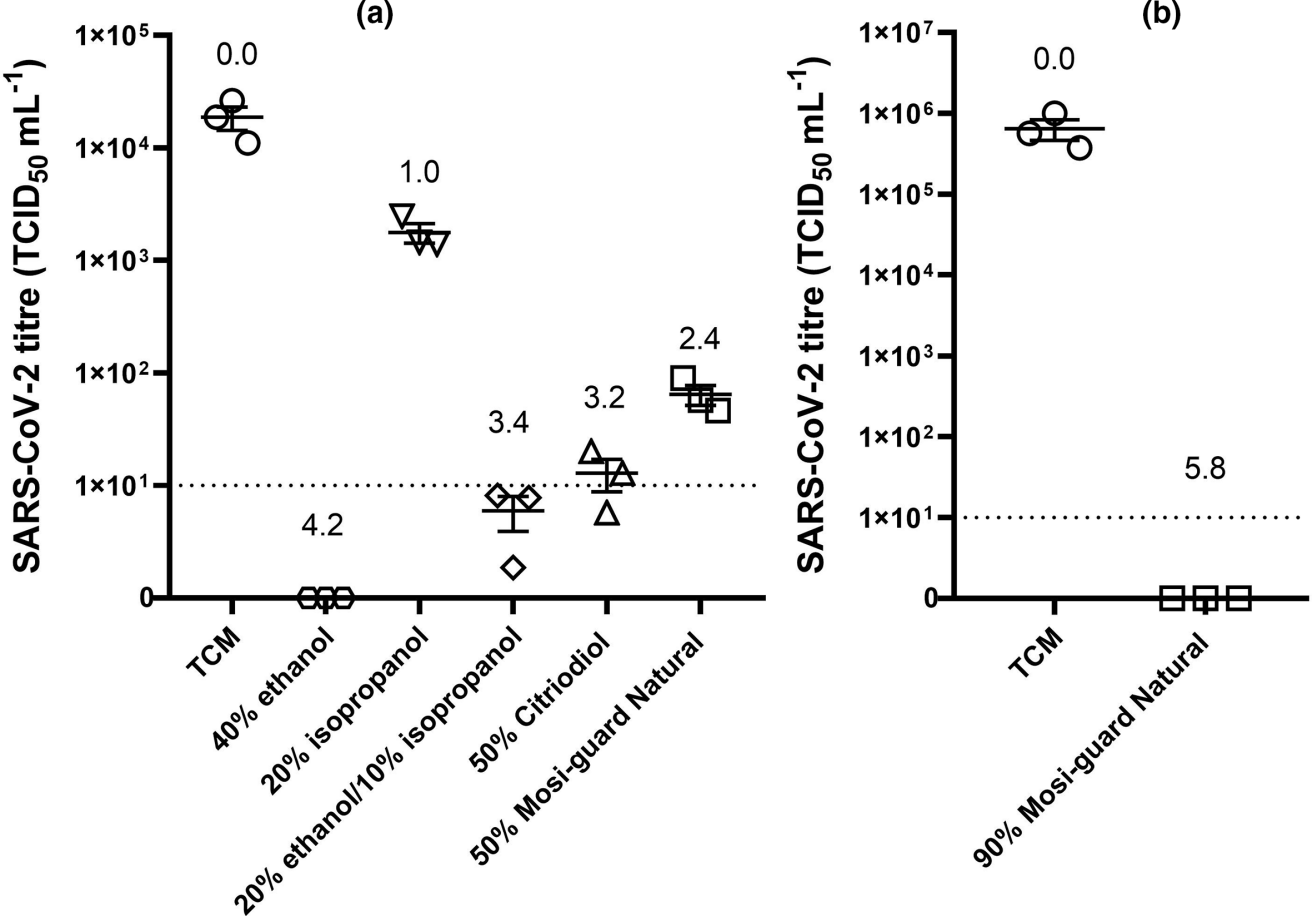
Virucidal activity of Mosi-guard Natural spray and its components. (a) In a standard measure of virucidal activity, SARS-CoV-2 was mixed 1 : 1 with Mosi-guard Natural spray, Citriodiol, ethanol, isopropanol or a combination of ethanol and isopropanol to give final concentrations as indicated on the x-axis. Viral titres of SARS-CoV-2 recovered after 1 min contact time are shown. (b) To measure Mosi-guard Natural spray at close to neat, concentrated SARS-CoV-2 was mixed 1 : 10 with Mosi-guard Natural spray to give a final concentration of 90 % (v/v). For both (a, b) each replicate is shown with a solid line at the geometric mean and error bars representing +/-sem. The dotted line at 10 TCID_50_ ml^−1^ is the limit of quantification of the assay. The number above each set of data points is the log_10_ reduction in viral titre observed.

In contrast, no viable virus was recovered by TCID_50_ assay or in flask passage when concentrated virus in TCM (mean initial titre of 6.45×10^5^ TCID_50_ ml^−1^) was mixed with 90 % Mosi-guard Natural (v/v) (Fig. 1b), resulting in a 5.8 log_10_ reduction in titre.

Statistical analysis using one-way ANOVA and Dunnett’s tests showed that all treatments tested gave a significant decrease in viral titre compared to the SARS-CoV-2 and TCM controls (*P*<0.001).

The activity of the insect repellent was examined in a first-of-its-kind protocol to reflect a possible use as barrier protection by topical application on skin. Latex, used as a synthetic skin, and an additional control surface (a polystyrene cell-culture plate) were treated with Mosi-guard Natural spray approximately 1 h before virus was applied. There was a notable difference on the wetting characteristics of insect repellent sprayed on the different surface types followed by addition of virus, which may have affected efficacy. The Mosi-guard Natural spray gave a relatively uniform coverage of liquid on the latex surface, and when virus was added it remained as discreet droplets. However, when applied to the polystyrene surface, an uneven distribution of Mosi-guard Natural spray was observed and the virus dispersed and wetted the whole surface of the well.

On both latex and plastic surfaces, there was minimal change in titre over 4 h on the untreated surfaces, but a decrease in titre, with immediate effect, on the Mosi-guard Natural-treated surfaces was observed (Fig. 2).

**Fig. 2.**
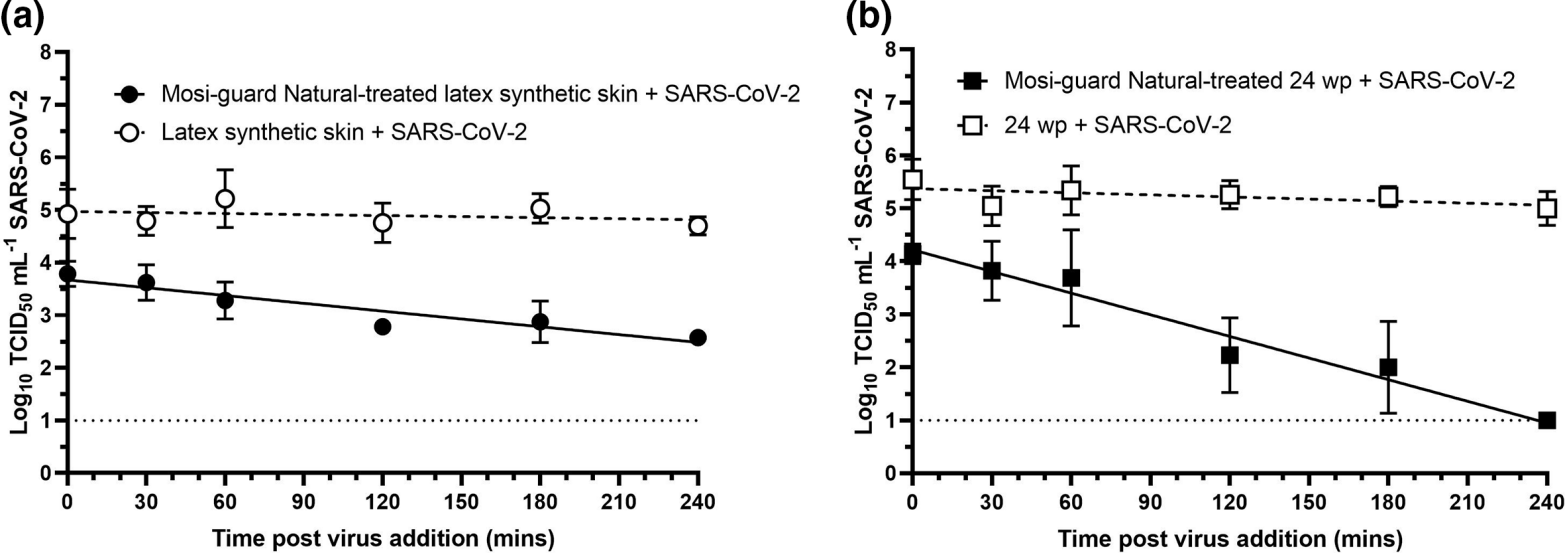
Change in viral titre over time on Mosi-guard Natural-treated or untreated surfaces. Latex synthetic skin discs (a, circles) or a polystyrene 24-well plate (b, squares) were treated with Mosi-guard Natural (filled shapes, solid lines) or left untreated (open shapes, dashed line). SARS-CoV-2 was added and recovered at various time-points. TCID_50_ counts were log_10_-transformed and linear regression performed. For both (a) and (b) each point represents the geometric mean of three replicate samples each assayed in duplicate with error bars representing +/-sem. The dotted line at 1 log_10_ TCID_50_ is the limit of quantification of the assay.

Recovery efficacy from the latex synthetic skin was lower than from the 24-well plate, but pre-application of Mosi-guard Natural spray to a latex synthetic skin resulted in a reduction of at least 1 log_10_ in viral titre of SARS-CoV-2 England-2 isolate (*P*<0.001) at all time-points and an approximate 1.5 log_10_ reduction after 4 h on the surface, however, virus was recoverable at all time points (Fig. 2a). On the Mosi-guard Natural-treated 24-well plate there was a greater reduction in viable virus that increased over time but viable virus could also still be recovered at every time-point (Fig. 2b). Linear regression analysis showed there was no measureable decay in untreated samples (*P*=0.547 for latex synthetic skin and *P*=0.113 for 24-well plate) whilst on both Mosi-guard Natural-treated surfaces decay was observed (*P*<0.001 for both).

A parallel experiment was carried out to estimate evaporation by weighing Mosi-guard Natural-treated latex sections over time. Up to 44 % total weight loss was measured but the mean weight loss was 10 % and the median weight loss was 12 % (data not shown). Most weight loss occurred within an hour of treatment (Data S1, available in the online version of this article); likely due to the more volatile compounds (alcohols) evaporating. There was no significant weight loss on the Mosi-guard Natural-treated surface after 1 h (Data S1). This suggests that there should be no solvent present that could affect the virology results and the non-volatile components of Mosi-guard Natural spray left on the latex surface would have remained constant throughout the duration of the parallel 4 h experiment with SARS-CoV-2 (Fig. 2).

Two studies were performed to assess the virucidal potential of an insect repellent against SARS-CoV-2. Firstly the insect repellent was tested at two different concentrations alongside its main constituents (Citriodiol, ethanol and isopropanol) in a standard test. Mosi-guard Natural spray (50 % v/v) and Citriodiol (50 % v/v) decreased viral titre by approximately 2 and 3 log_10_ respectively, but not below the limit of quantification. At a higher concentration (90 % v/v), Mosi-guard Natural spray gave a significant decrease of over 4 log_10_ resulting in no recoverable virus. Unsurprisingly, ethanol alone (used at 40 %) had virucidal activity in our assay, and when used at 20 % in combination with isopropanol. Efficacy of ethanol against SARS-CoV-2 has already been reported [7, 8]. In our test, isopropanol used at 20 % (v/v) was found to have limited efficacy but the concentration was low; experiments have shown isopropanol should be used at 30 % or greater to have virucidal activity [8, 9]. To date, there have been limited antimicrobial activity studies with essential oils for comparison with our Citriodiol data. Essential oils have been shown to have activity against Influenza virus [10, 11] and the essential oils of Eucalyptus globulus have also been shown to have antibacterial activity [12].

We have shown that Mosi-guard Natural spray, which is intended to be sprayed onto skin to repel insects, also has potential to reduce the level of SARS-CoV-2 on exposed skin. This was done to test the potential for topical application of insect repellent as a potential prophylactic barrier against contact transmission or exposure to droplets. This was the first time we have performed such an experiment looking at the survival of virus on an impregnated surface. Significantly less SARS-CoV-2 virus was recovered from Mosi-guard Natural-treated surfaces compared to untreated surfaces, but viable virus was still recovered. The methodology developed during this study could be extended to other types of surfaces and/or other compounds with virucidal activity.

There were limitations to our study, particularly in the treated surface experiments where we tried to replicate how the insect repellent might have virucidal activity when on human skin. There are a number of further experiments that could be performed. We used latex as a synthetic skin at room temperature (19–22 °C), which is lower than the temperature at the surface of human skin. A study using pig skin determined a half-life of SARS-CoV-2 of 3.5 h at 22 °C and recovered virus at 96 h, but at 37 °C viable virus was detected at 8 h but not 24 h [13]. On autopsy specimens of human skin the virus was shown to survive for 9 h at 25 °C and 45–55 % RH [14]. By performing our study at room temperature we had a consistent amount of viable virus over time as there was minimal natural decay. In both experiments, virus was applied suspended in tissue culture media. SARS-CoV-2 suspended in artificial saliva or other bodily fluids survives less well than in TCM [unpublished personal observations; also reported by 14, 15] and so we consider that we used a ‘worse-case’ scenario that provides increased confidence in the results. Using TCM, rather than diluting into another matrix, also allowed work to be done with higher titres of virus. Virus was added in discreet droplets onto the synthetic skin but in reality droplets from a sneeze or cough will vary in size and might disperse upon impact onto skin. In our study virus was added at a single time-point approximately 1 h post-treatment of the latex skin substitute: we did not investigate whether ageing the insect repellent treatment prior to virus exposure would affect the virucidal activity. Our results however, provide some confidence that Mosi-guard Natural spray maintains virucidal activity over a short time on a latex surface and we have shown, in the liquid assays, that Citriodiol as well as the alcohol components of Mosi-guard Natural spray had some individual level of virucidal activity. Our estimation of evaporation by measuring weight loss on treated-surfaces seemed to back this up with negligible weight loss on Mosi-guard Natural-treated latex after an hour. We hypothesise this was due to the alcohol content evaporating but further work would be needed to confirm this.

In conclusion we have shown that in a 1 min liquid suspension test Mosi-guard Natural spray has virucidal activity against SARS-CoV-2 England-2 isolate. Additionally, viral studies on latex indicated that the insect repellent also has virucidal activity against SARS-CoV-2 on a Mosi-guard Natural-treated surface used to simulate skin. This offers the potential that topical application of the insect repellent could have some prophylactic potential. These small-scale studies can inform the basis of further research in this area.

## Supplementary Data

Supplementary material 1
