# Supplementary material for: Investigative study into whether an insect repellent has virucidal activity against SARS-CoV-2
Source: J Gen Virol. 2021 Apr 23;102(4):001585. doi: 10.1099/jgv.0.001585 (PMC8290268; doi:10.1099/jgv.0.001585)
Supplement: Supplementary material 1 [file jgv-102-1585-s001.pdf]

Supplementary Data

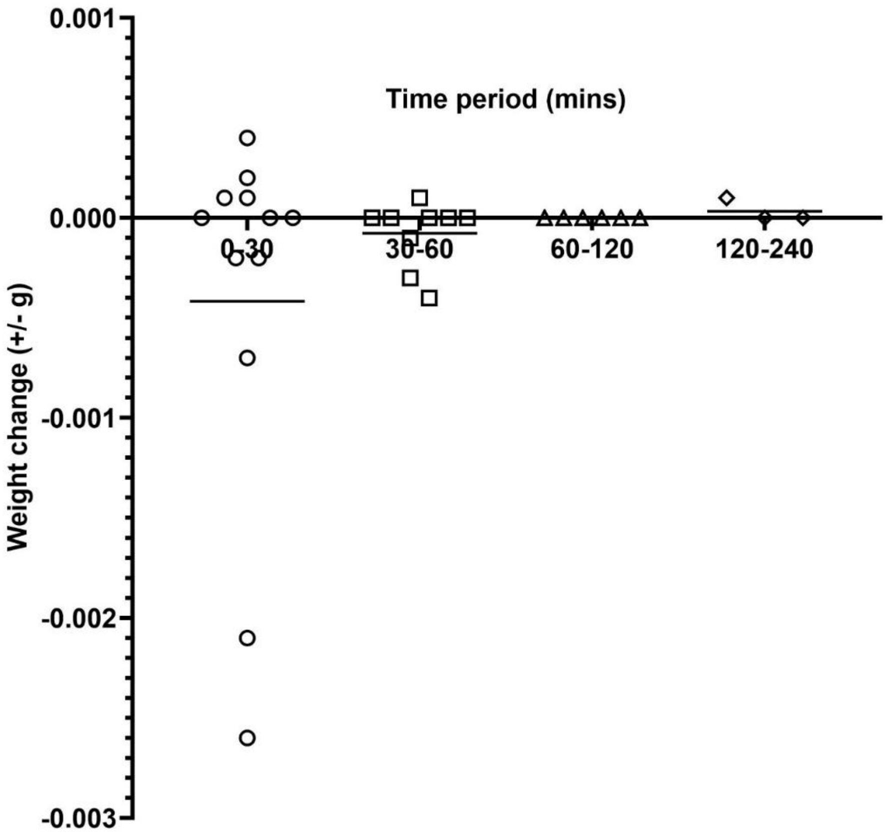

Estimation of Mosi-guard Natural® spray evaporation by weight loss. 12 discs of latex synthetic skin were treated with Mosi-guard Natural® spray. The t=0 weights were determined for all 12 discs. Weights were then determined after 30, 60, 120 and 240 minutes. At these time points three discs were removed and weighed. The weight gain (above x-axis) or loss (below x-axis) in grams during the time periods is shown with a line at the mean.
